# Tribute to Andrea Huwiler (1966–2023)

**DOI:** 10.1007/s00424-024-02958-5

**Published:** 2024-04-09

**Authors:** Josef Pfeilschifter, Erich Gulbins

**Affiliations:** 1 Institut für Allgemeine Pharmakologie und Toxikologie, Goethe-Universität, Frankfurt Am Main, Germany; 2 Institut für Molekularbiologie, Universitätsklinikum Essen, Essen, Germany


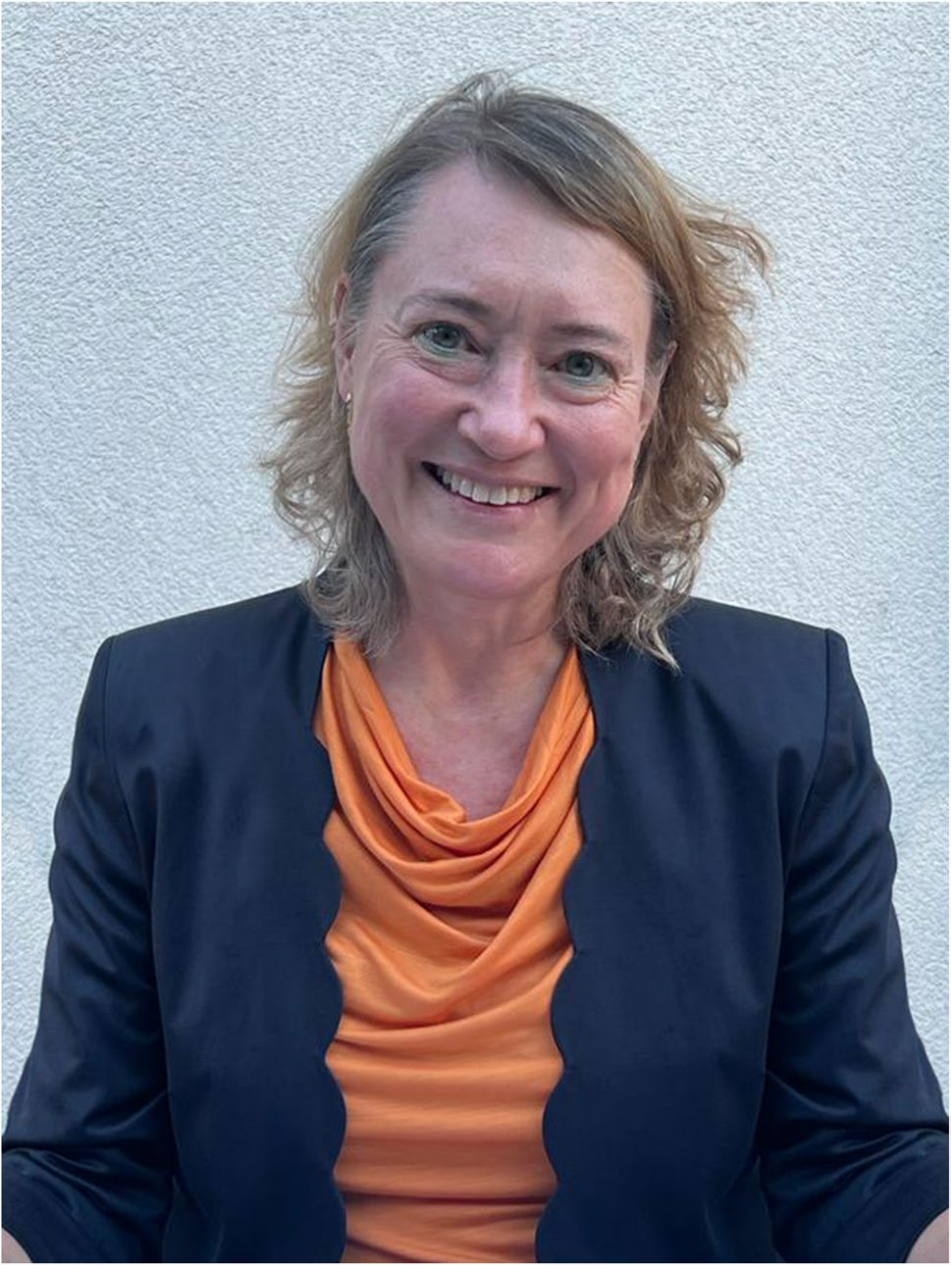

“I feel,” she said after a busy day, “suddenly very, very unwell,” became pale, and sat down.

Just shortly before, in the laboratory, pipetting, measuring, experimenting, she had been in an almost euphoric mood, which she openly displayed—for she was actually an introverted, self-effacing person, sometimes almost shy, expressive and dramatic performances were not her thing.

“I think,” she said, “I need a doctor.”

A doctor, many doctors, many very good doctors were quickly on hand, because the laboratory and the Pharmacological Institute, of which she was the director, are located in the main building of the Inselspital in Bern—and that is the local university hospital. But even the combined surgical and intensive medical power of a university hospital, all the helping hands, could not wrest her from death, which had already firmly grasped her hand. She died on December 18, 2023, a few days after she had sat down, not even 58 years after she had come into the world. Suddenly and unexpectedly, for her, for us. A lightning bolt from the clear sky of an early afternoon, an unexpected tear in her thread of life, which one had imagined spun more solidly and inclined to less dramatic tears.

She was the child of a Norwegian mother and a Swiss father and was born on September 22, 1966, in Drammen, Norway. She grew up in Norway and Switzerland. She studied biochemistry at ETH Zurich and graduated in 1990 with a diploma in natural sciences. In the Inflammation and Allergy Department of Ciba-Geigy in Basel, she received her Ph.D. in pharmacy from the University of Basel in 1993, with a thesis on the regulatory functions of protein kinase C isoforms. She continued her research as a postdoctoral fellow in the laboratory of Professor Josef Pfeilschifter at the Biozentrum of the University of Basel (1993–1996). Two further research stays took Andrea Huwiler to the laboratory of Professor Edward Dennis at the Department of Biochemistry at UCSD in La Jolla (1996–1997) and to Professor Henk van den Bosch at the Center for Biomembranes and Lipid Enzymology (CBLE) in Utrecht (1997–1998). In 1998, Andrea Huwiler took over the leadership of an independent research group at the Institute of General Pharmacology and Toxicology at Goethe University in Frankfurt am Main. There she was habilitated in 1999 for the field of Biochemical Pharmacology and appointed as a university lecturer (C2) in 2001. In 2005, she was appointed as an extraordinary professor at the Faculty of Medicine of Goethe University. In 2006, three parallel calls for professorships were made to the Universities of Dresden, Bern, and Frankfurt am Main, and Andrea Huwiler decided to return to Switzerland. In 2006, she became an Extraordinaria at the Pharmacological Institute of the University of Bern, which she also headed as director since 2021.

Andrea Huwiler was a gifted scientist. With great enthusiasm, she dealt with lipids at a time when lipids were far from being in the focus of the scientific community. She steadfastly worked on elucidating the mechanisms of action of ceramide and sphingosine-1-phosphate and witnessed how under the slogan “Lipid Signaling” this area of research returned to the main stage of interest for biochemists, physiologists, pharmacologists, and clinicians. Her pioneering work established relevant pathophysiological functions for sphingosine kinases in kidney diseases and fibrotic diseases in general. She also transferred these findings into the development of corresponding drug candidates, some of which are already in clinical trials. Her work was honored with numerous awards, including the Emil Bürgi Prize from the University of Bern in 1994, the Novartis Pharmacology Prize for Young Scientists in 1997, the Franz Volhard Prize from the German Society of Nephrology (DGfN) in 2004, and the British Pharmacological Society Award in 2019. For the DGfN, she co-chaired the Physiology and Pharmacology Commission with Professor Richard Warth.

Andrea Huwiler was also an inspiring academic teacher. She exerted a strong fascination on the people around her, whom she could inspire with her scientific ideas. She had the ability to present highly complex relationships in an understandable, comprehensible, and interesting way, and her teaching activities were highly valued. We remember Andrea Huwiler as an exceptional scientist, pharmacologist, companion, teacher, mentor, role model, and friend. We are grateful for what she has given us on our way, for her inspirations as well as for her constructive criticism.

Andrea Huwiler worked on projects that she always completed. She was not a fan of half measures. Whether she also regarded her life as such a “project,” to which death—which also does not do things by halves—has now put an end, we do not know. We, however, stand admirably before her life’s work. We will remember her for a long time and mourn together with her loved ones.

She will be buried where she was born, in Drammen. This is a city in southern Norway and, as befits Norway, it is at the end of a fjord. But the Drammen Fjord is not a wild coastal inlet framed by dramatic cliffs and treacherous mountains full of trolls—it is a hilly landscape full of rivers, forests, and fields, which surround the fjord. Quite unexcitedly, an introverted, unobtrusive landscape that one learns to appreciate and love when one engages with it. That fits because we felt no differently about her.

## Data Availability

No datasets were generated or analysed during the current study.

